# Spectral characteristics of gold nanoparticle doped optical fibre under axial strain

**DOI:** 10.1038/s41598-022-20726-2

**Published:** 2022-10-05

**Authors:** Xiang Wang, Rinze Benedictus, Roger M. Groves

**Affiliations:** 1 Faculty of Aerospace Engineering, Delft University of Technology, 2629 HS Delft, The Netherlands; 2 Aerospace Non-Destructive Testing Laboratory, Delft University of Technology, 2629 HS Delft, The Netherlands

**Keywords:** Imaging and sensing, Aerospace engineering, Nanoparticles, Nanoparticles

## Abstract

Nanoparticle (NP) doping of optical fibres can be used to increase the intensity of the backscattered light used for distributed strain sensing and has shown the advantages of high precision strain detection and multiplex sensing experimentally. However, the backscatter spectral characteristics of NP-doped optical fibres have not been described even though they are quite different from the spectra from fibre Bragg gratings (FBGs) or commercial single mode fibres. In this paper, gold NPs, used as the contrast agent in the optical fibre to increase the intensity of the backscattered light, were investigated from the aspect of their spectra. A single scattering model with Mie theory and an effective refractive index (RI) model were used to evaluate the backscattered light spectra and the Monte Carlo Method was used for seeding NPs. The results showed that the strain responsivity of gold-NP doped fibres with low volume ratio doping (single scattering restriction) are close to FBGs and commercial fibres. High volume ratios of gold NP doping increase the imaginary part of the RI of the optical fibre, which has a significant influence on the spectra in the wavenumber domain. These theoretical insights may promote the future engineering design of NP-doped fibre sensors.

## Introduction

The behaviour of the spectra shift under strain for optical fibre and that it is similar to that from fibre Bragg gratings (FBGs) has been demonstrated and has been used for strain sensing since the 1990s^1^. A refractive index (RI) fluctuation in the optical fibre causes this scattered light in the optical fibres^2,3^. Backscatter reflectometry uses the backscattered light and by using cross-correlation of the spectra before and after strain changes and auto-correlation with spectra before strain change, the wavelength shift can be obtained^4^. This wavelength shift can then be used to obtain strain or temperature information based on the responsivity of the sensors. For a commercial optical fibre, Rayleigh scattering is the major component for light scattering. This Rayleigh scattering based distributed fibre optic sensing has the advantages of long distance sensing, low weight, immunity to electromagnetic interference etc. and has been widely used in a variety of fields, such as aerospace engineering^5^ and civil engineering^6^ for structural health monitoring (SHM).

Rayleigh scattering based distributed fibre optic sensing usually uses commercial communication optical fibres as the sensing components. The optical fibres used for communication are generally low loss and low dispersion fibres in the communication wavelength bands. One reason for using communication optical fibres as the sensing fibres is that they can achieve long distance sensing, for example several kilometres, due to their low attenuation (about 0.2 dB/km @ 1550 nm). Meanwhile, they are also relatively cheap due to mass production for the communication industry. Therefore, they are often used as the sensors for distributed fibre optic sensing. However, the backscattered light signal in the communication optical fibre is generally very low (− 100 dB/mm for single mode fibre (SMF)^7^), which has become a limitation of the Rayleigh scattering based technique^8^ for some applications. For SHM of aircraft structures, only key parts for example the areas close to the rivet holes usually need to be monitored and the required distance is not necessarily very long but high spatial precision is required. In this case long distance sensing may not be an advantage. To address this, it is necessary to enhance the local scattered light signal near the key parts of the aircraft structures to improve the signal-to-noise ratio to get a better monitoring of the key parts.

In order to increase the intensity of the backscattered light signal in the optical fibres, several methods have been proposed. The ultraviolet (UV) light irradiation method can greatly increase the intensity of the scattered signal by about 20 dB, which may be due to the numerical aperture increase after exposure^8^. This method has also been used in surgery applications^9^. Femtosecond lasers can generate microstructures in the core of the optical fibres^10,11^. The random microstructures improve the backscattered signal dramatically which also improves the signal to noise ratio for distributed fibre optic sensing. Both of these methods are based on laser damage of the optical fibres.

NPs have been widely used to pump the light signal in optical fibres^12,13^. The RI difference of the NPs to the core of the optical fibre will also cause the backscattered light in the optical fibre. Therefore, doping NPs into the core of the fibres is an approach to achieving backscattered light enhancement. In addition, as the size of the NPs increases, the Rayleigh scattering in the optical fibre will tend to Mie scattering when the size of the NP is comparable with the incident light^14^ and this causes a dramatic light intensity increase. For this reason, in recent years, NP-doped optical fibres have been investigated to overcome the low scattering limitation of the optical fibre and it has become an active research field.

Several oxide or metallic materials have been chosen as the scattering agents to increase the scattered light. As an oxide material, magnesium oxide (MgO) has been studied extensively by researchers because MgO has low attenuation and also has the ability to increase the backscattered light in the optical fibres, which is required for long distance enhanced sensing^15^. By changing the temperature during manufacturing of optical fibre containing MgO, high scattering MgO-doped optical fibre has been achieved^16^. The relationship between the temperature and the backscattered light spectra wavelength shift has been studied experimentally. There are also some applications with the MgO-doped optical fibres, for example multiplexed sensing^17^.

Gold is one of the metallic materials that attracts interest in recent years especially for bio-imaging enhancement because of its highly scattering characteristics^18,19^. Gold NP doped optical fibres have the potential to increase the scattered light dramatically within a short distance (several centimetres to metres), so it may be suitable for short distance strain sensing for key areas which need to be monitored^20,21^. In experiment, zirconia-coated gold doped optical fibre has been investigated and showed an increase of stability for high temperature sensing (800)^22^.

However, there are few theoretical studies on the characteristics of the scattering spectra of NP doped optical fibres. A theoretical investigation is important to guide the engineering design of NP doped fibre sensors. In this paper, the characteristics of NP doped optical fibres will be investigated from the characteristics of their backscattering spectra. First, the characteristic of the spectra shift under strain will be analysed theoretically and then compared with the FBGs and commercial fibres cases. The nonlinearity between strain and wavelength shift caused by the introduction of NPs is analysed. Taking gold NP optical fibre as an example, the random fringe characteristics of the scattering spectra of gold NPs of different sizes and volume ratios are studied in the wavenumber domain.

## Theory

**Figure 1:**
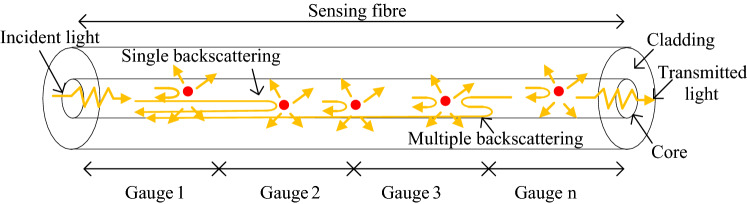
A schematic of the theoretical model. The red dots show the gold nanoparticles in the core of the sensing fibre in random positions.

A schematic of the theoretical model is shown in Fig. 1. The incident light from the tunable laser propagates along the sensing fibre. The incident light will be scattered and absorbed by the gold NPs in the core of the optical fibre. The transmitted light and forward and backward scattered light that can meet the requirements for transmitting in the optical fibre will propagates in forward and backward directions. The backscattered light and the forward scattered light can be scattered many times. The total collected backscattered light is a combination of the single backscattering and multiple backscattering. For the sensing fibre, it has been divided to *n* gauges. For simplification, the first, the second, the third, and the *n*th gauges are shown in the figure. Each gauge is used as a strain gauge for strain detection. When the axial strain changes, the relative positions of the NPs will change and the RI of the optical fibre will also change. Then, the backscattered light spectrum will shift. By demodulating the spectral shift, the strain values can be obtained.

To determine the backscattered spectra in the sensing path and to avoid time-consuming calculations, some assumptions are made. The NPs inside of the core of the optical fibres are assumed to be homogeneous spherical NPs with low volume ratios which meet the single scattering approximation as will be shown later, therefore the light scattered by the NPs can be calculated by Mie theory and only single backscattered light needs to be considered. A random distribution of the NPs in the optical fibre is assumed and the positions of the NPs were obtained by the Monte Carlo Method for seeding the NPs. In this case, the core of the optical fibre consists of two materials, fused silica fibre and gold NPs, so the RI of the core of the optical fibre needs to use the effective RI. In additon, for the backscattered light, only the light which is within the aperture angle can transmit backward in the optical fibre. Therefore, the backscattered light in the fibre in a sensing gauge length can be expressed as:

1 $$\begin{aligned} E_s=\sum _{i=1}^{N}\overline{\overline{r}}(l_i)E_i(l_i)\exp {j(k n_{eff}l_i)}, \end{aligned}$$

where, $E_s$ is the electrical field of the scattered light at the beginning of the gauge length, $E_i$ is the electrical field of the transmitted light at position $l_i$, *N* is the total number of the NPs in the gauge length. $\overline{\overline{r}}(l_i)$ is the complex reflectively of each NP at the *i*th NP’s position $l_i$, *j* is the imaginary unit and $n_{eff}$ is the effective RI of the core of the fibre. $k=2\pi /\lambda$ is the angular wavenumber in vaccum and $\lambda$ is the wavelength in vaccum. The electrical field of the transmission light at position $l_i$ can be expressed as:

2 $$\begin{aligned} E_i=E_0\exp {(j k n_{eff}l_i)}, \end{aligned}$$

where, $E_0$ is the electrical field of the incident light. The complex reflectively of each NP can be expressed as:

3 $$\begin{aligned} \overline{\overline{r}}(l_i)=r_i\exp {j\phi _i}\approx \frac{E_s(l_i)\sqrt{\Omega r^2}}{E_i(l_i)\sqrt{\pi R^2}}=\frac{\sqrt{\Omega }S_2(\pi )}{-jkn_{eff}\sqrt{\pi } R}, \end{aligned}$$

where, $\Omega$ is the solid angle which corresponds to the aperture angle, *r* is the distance to the centre of each NP, *R* is the radius of the core region of the fibre, and $S_2(\pi )$ is the scattering parameter at a backward angle $\pi$. Then, the amplitude and phase of the complex reflectively can be expressed as:

4 $$\begin{aligned} r_i=|\overline{\overline{r}}(l_i)| \end{aligned}$$

and

5 $$\begin{aligned} \phi _i=\angle \overline{\overline{r}}(l_i). \end{aligned}$$

The relationship between the incident light and the scattered light can be expressed as:

6 $$\begin{aligned} \left[ \begin{array}{c} E_{\parallel s}\\ E_{\perp s} \end{array} \right] = \frac{\exp {jkn_{eff}(r-z)}}{-jkn_{eff}r}\left[ \begin{array}{cc} S_2(\mu ) &{} 0\\ 0 &{} S_1(\mu ) \end{array} \right] \left[ \begin{array}{c} E_{\parallel i}\\ E_{\perp i} \end{array} \right] , \end{aligned}$$

where, $E_{\parallel i}$ and $E_{\perp i}$ are the two orthogonal components of the incident light, $E_{\parallel s}$ and $E_{\perp s}$ are the two orthogonal components of the scattered light with angle $\mu$, $\mu =\cos {\theta }$ and $\mu =-1$ for backward scattering. The transmitted light in the optical fibre is assumed to be polarised light for simplification. For a more general case, the light can be decomposed to a combination of the parallel component and the perpendicular component but this was not taken into consideration in this paper.

The van de Hulst approximation has been used for calculation of the effective RI of gold NP-doped MoO$_3$ film^23^ when the system is considered as a dilute turbid system. When the single scattering approximation is considered, the volume ratio of gold NP in the optical fibre is low. Then, it is assumed that the effective RI of NP-doped optical fibre can be expressed according to the van de Hulst approximation for low volume ratio doping based on the equation from Gesuri Morales-Luna and Michael Morales-Luna^23^ as:

7 $$\begin{aligned} n_{eff}=n_m\left( 1+j\frac{3 f S(0)}{2x^3}\right) , \end{aligned}$$

where, $n_m$ is the RI of the optical fibre (fused silica), *f* is the volume ratio of the NPs in the core of optical fibre, *S*(0) is the forward scattering amplitude which can be calculated by Mie theory^14^ and the RI of gold used for calculation is based on the size dependant RI of gold^21,24^. *x* is the size parameter of the NP in the optical fibre.

Then, the electrical field for backscattered light can be expressed as:

8 $$\begin{aligned} E_s =\sum _{i=1}^{N}E_0 A_i \exp {j\left( \frac{4\pi n_m l_i}{\lambda } \left( 1-\frac{3 f \Im S(0) }{2x^3}\right) +\phi _i\right) }, \end{aligned}$$

where, $$A_i=r_i\exp {\left( -6\pi f n_m \Re S(0) l_i/\lambda x^3 \right) }$$.

If the NP inside the optical fibre can be assumed to be an isotropic material and the axial strain of the optical fibre changes, the volume of the NP may change from sphere to spheroid. According to the geometry, the volume ratio after strain change can be expressed as:

9 $$\begin{aligned} f'=\frac{(1+\varepsilon _{gold})(1+\gamma _{gold}\varepsilon _{gold})^2}{(1+\varepsilon _{fibre}) (1+\gamma _{fibre}\varepsilon _{fibre})^2}f, \end{aligned}$$

where, $\varepsilon _{gold}$ is the axial strain of the gold NPs, $\varepsilon _{fibre}$ is the axial strain of the optical fibre and $\gamma _{gold}$ is the ratio of transverse strain to axial strain of the gold NPs, $\gamma _{fibre}$ is the ratio of transverse strain to axial strain of the optical fibre, *f* is the volume ratio of gold NPs to the optical fibre before strain change and $f'$ is the volume ratio of gold NPs to the optical fibre before strain change.

The relative axial strain changes between the NP and the optical fibre ($\alpha$) is defined as $\alpha =\varepsilon _{gold}/\varepsilon _{fibre}$. Then, the electrical field of the scattered light in the sensing fibre can be expressed as:

10 $$\begin{aligned} E_s(\varepsilon _{fibre})=\sum _{i=1}^{N}E_0 A_i \exp {j\left( \frac{4\pi n_m'(\varepsilon _{fibre}) l_i'(\varepsilon _{fibre})}{\lambda } \left( 1-\frac{3 f \Im S'(0) }{2x'^3}\right) +\phi _i\right) }, \end{aligned}$$

where,

11 $$\begin{aligned} n_m'(\varepsilon _{fibre})=n_m(1+\eta _m \varepsilon _{fibre}), \end{aligned}$$

and

12 $$\begin{aligned} l_i'(\varepsilon _{fibre})=l_i(1+\varepsilon _{fibre}). \end{aligned}$$

The size parameter for the spherical NP is defined as $x=2\pi r_{NP}/\lambda$, where $r_{NP}$ is the radius of the sphere. According to the geometry change of the NP under strain, the geometric cross section will be expanded or compressed in the case of axial strain is negative (compression) or positive (tension). Therefore, the size parameter of the NP under strain can be expressed as:

13 $$\begin{aligned} x'=\frac{n_m'}{n_m}x(1+\alpha \varepsilon _{fibre}). \end{aligned}$$

If the forward scattering amplitude *S*(0) is assumed to follow a linear relationship under strain, then the forward scattering amplitude $S'(0)$ can be expressed as:

14 $$\begin{aligned} \Im S'(0)=\Im S(0)(1+\gamma _S \varepsilon _{fibre}). \end{aligned}$$

Therefore,

15 $$\begin{aligned} E_s(\varepsilon _{fibre})\approx \sum _{i=1}^{N}E_0 A_i\exp {j\left( \frac{4\pi n_m l_i}{\lambda } K (1+\varepsilon _{fibre})(1+\eta _m \varepsilon _{fibre}) +\phi _i\right) }. \end{aligned}$$

Using a first order Taylor expression at a strain value of zero,

16 $$\begin{aligned} \begin{aligned} K&\approx 1-\frac{3f \Im S(0)}{2x^3} K', \end{aligned} \end{aligned}$$

where,

17 $$\begin{aligned} K'= 1+(\alpha -\alpha \gamma _{gold} +\gamma _S -1-2\gamma _{fibre}-3\eta _m)\varepsilon _{fibre}. \end{aligned}$$

Therefore, the characteristic peaks also change their wavelengths according to

18 $$\begin{aligned} \lambda '_{NP}=\lambda _{NP} K(1+\varepsilon _{fibre})(1+\eta _m\varepsilon _{fibre}) \approx \lambda K(1+\varepsilon _{fibre}+\eta _m\varepsilon _{fibre}) \end{aligned}$$

Equation (18) is similar to the expression for a FBG, which can be expressed as $\lambda _B=2n\Lambda$, where, *n* is the effective RI of the FBG and $\Lambda$ is the grating period. When the strain changes, $\Lambda$ and *n* will change and the expression for the FBG under strain can be expressed as

19 $$\begin{aligned} \lambda '_B=\lambda _B (1+\varepsilon _{fibre})(1+\eta _m\varepsilon _{fibre}) \approx \lambda _B (1+\varepsilon _{fibre}+\eta _m\varepsilon _{fibre}), \end{aligned}$$

where, $\lambda _B'$ and $\lambda _B$ are the Bragg wavelength of the FBG with and without strain change.

For Rayleigh scattering based distributed fibre optic sensing, the wavelengths under strain can be expressed as^25^

20 $$\begin{aligned} \lambda '_R= & {} \lambda _R (1+\varepsilon _{fibre}+\eta _m\varepsilon _{fibre}) \end{aligned}$$

21 $$\begin{aligned} \eta _m= & {} -\frac{p_{12}-v(p_{11}+p_{12})}{2}n^2 \end{aligned}$$

where, $p_{11}=0.113$, $p_{12}=0.252$, *v* is the Poisson’s ratio of optical fibre and $v=0.17$. If the RI is set at 1.45, then $\eta _{m}=-0.1997$.

In order to make a comparison between the Bragg wavelength shift of a FBG, the wavelength shift of distributed fibre optic sensing based on Rayleigh scattering and the spectral wavelength shift in the NP doped optical fibre, the expressions are summarised in Table 1.

**Table 1:** The expressions comparison among different types of fibres.

Type	Expression
Fibre Bragg grating	$\lambda '_B=\lambda _B (1+\varepsilon _{fibre}+\eta _m\varepsilon _{fibre})$
Distributed sensing based on Rayleigh scattering	$\lambda '_R=\lambda _R (1+\varepsilon _{fibre}+\eta _m\varepsilon _{fibre})$
NP doped optical fibre	$\lambda '_{NP}=K\lambda _{NP} (1+\varepsilon _{fibre}+\eta _m\varepsilon _{fibre})$

It can be seen from Table 1 that for a FBG, distributed sensing based on Rayleigh scattering and a NP doped optical fibre, the wavelength shift expressions are in similar forms so now the sensitivities can be easily compared. However, for Bragg grating there is a Bragg wavelength while for the distributed sensing based on Rayleigh scattering and NP doped optical fibre there are random peaks. In addition, for a NP doped optical fibre, the wavelength shift also be influenced by a factor of *K* which is a nonlinear contribution to the responsivity caused by NP doping.

## Results and discussion

**Figure 2:**
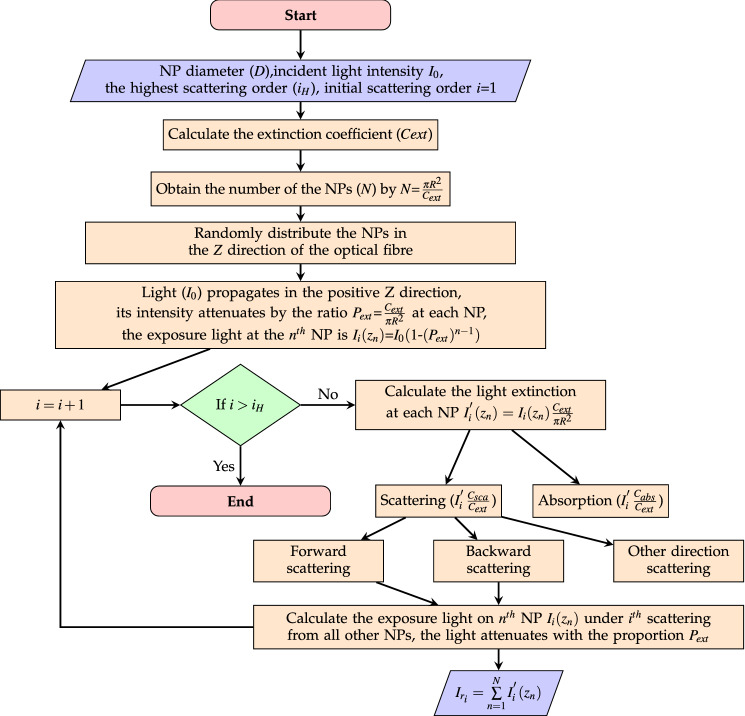
Flowchart of up to *N*th order scattering received from an optical fibre input port.

**Figure 3:**
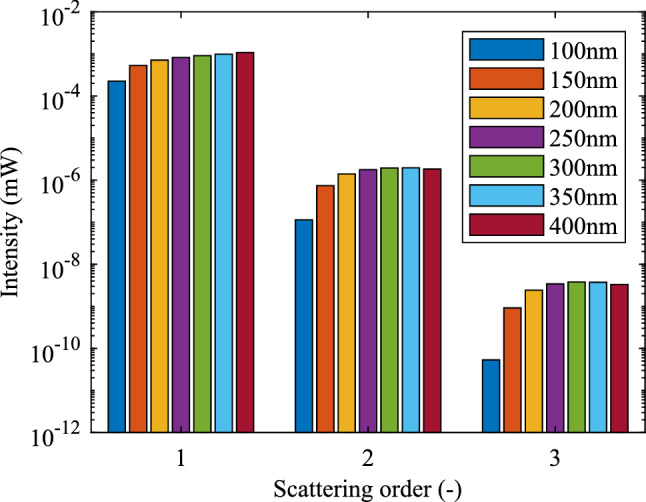
Light intensity under different scattering orders ($1 \hbox {mW}$ incident light).

Light could be scattered multiple times in the optical fibre by the NPs, but the higher order scattering (scattered more than one time) shows a low light intensity. In order to show the scattered light intensity received at the incident port which is within the single scattering length ($z=1/cC_{ext}$, where *c* is the concentration of the NPs and $C_{ext}$ is the extinction cross section of the NP), the flowchart shown in Fig. 2 is used for this calculation. The flowchart begins by checking if the order of the scattering (*i*) is larger than the set highest scattering order ($i_H$). The results of the intensities of different scattering order are shown in Fig. 3. The incident light power is set to 1 mW.

Single scattering components are much larger than the higher order scattering shown in Fig. 3. The intensities of twice scattering (scattering order 2) are 0.0505 %, 0.3205 %, 0.1962 %, 0.2136 %, 0.1984 % and 0.1697 % compared with single scattering (scattering order 1) for 100 nm, 150 nm, 200 nm, 250 nm, 300 nm, 350 nm and 400 nm size gold NPs respectively. It can be seen that the components for multiple scattering is low. Therefore, only considering the single scattering is suitable for the calculation and can reduce the calculation time.

**Figure 4:**
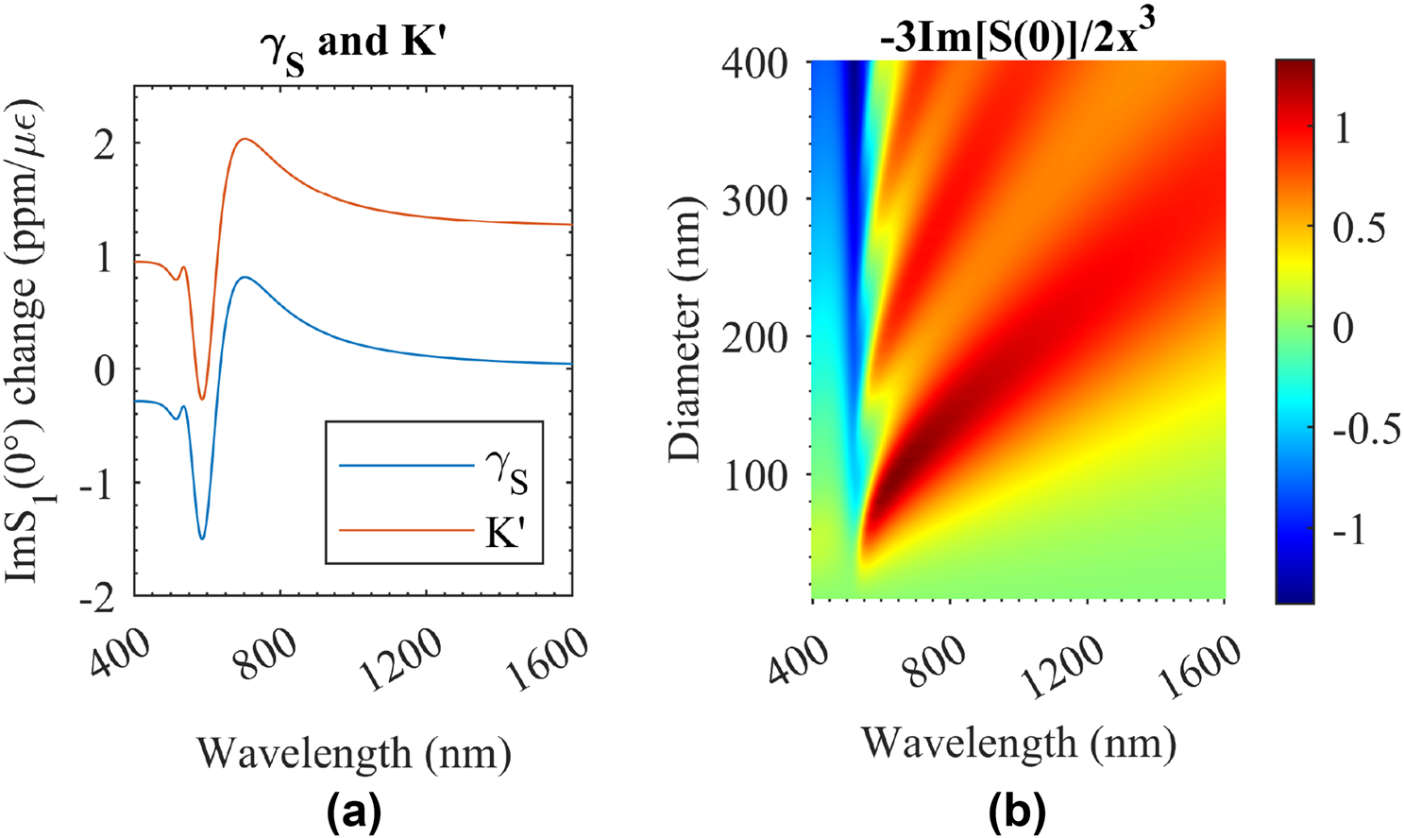
The nonlinear factors. (**a**) $\gamma _S$ and $K'$; (**b**) The coefficient before $K'$.

The difference between the expressions of the spectral shift of NP doped optical fibre and FBG or distributed fibre optic sensing based on Rayleigh scattering is that there is an additional nonlinear factor of K (see Table 1). It can be seen from Eqs. (16) and (17) that the coefficient before $K'$ determines the deviation from the other two cases and $K'$ varies with $\gamma _S$ and $\eta _m$ under different wavelengths. $\alpha$, $\gamma _{gold}$, $\gamma _{fibre}$ are determined by the mechanical properties of the materials. For the optical fibre with 100 nm size gold NPs with $\alpha =0.92$, $\gamma _{fibre}=-0.17$, $\gamma _{gold}=-0.40$, $\gamma _S$ can be calculated and is shown in Fig. 4a. If $\eta _m$ is assumed to be a constant, then $K'$ can be calculated and is shown in Fig. 4. Although $K'$ varies a lot in the wavelength range, the strain applied to the optical fibre is a small strain (less than several ${0.001} {\epsilon }$). Therefore, $K'$ has a negligible effect on *K*. The coeffiecent before $K'$ is shown in Fig. 4b and it is quite small when it multiplies a low volume ratio (*f*). If $f=0.001$ is used, it is a large volume ratio so the single scattering assumption maybe not effective. Even for this case, the parameter *K* is almost at 1 with small variation (less than 0.2 %) within the spectra range from 400 to 1600 nm and the NP diameter range from 10 to 400 nm, so the nonlinear property of the nonlinear factor *K* is quite small when the volume ratio of the NPs in the optical fibre is small which is in this case $K\approx 1$. Therefore, for the following case study of different sizes and volume ratios of NPs in different gauge lengths, *K* is set to 1 for simplification. Then, the characteristic spectra were obtained using Eq. (15). The wavelength range was from 1545 to 1555 nm and the wavelength resolution used for simulation was 1 pm. The strain was set at 100 με. The gold NPs were seeded in the specific gauge lengths with their concentrations.

**Figure 5:**
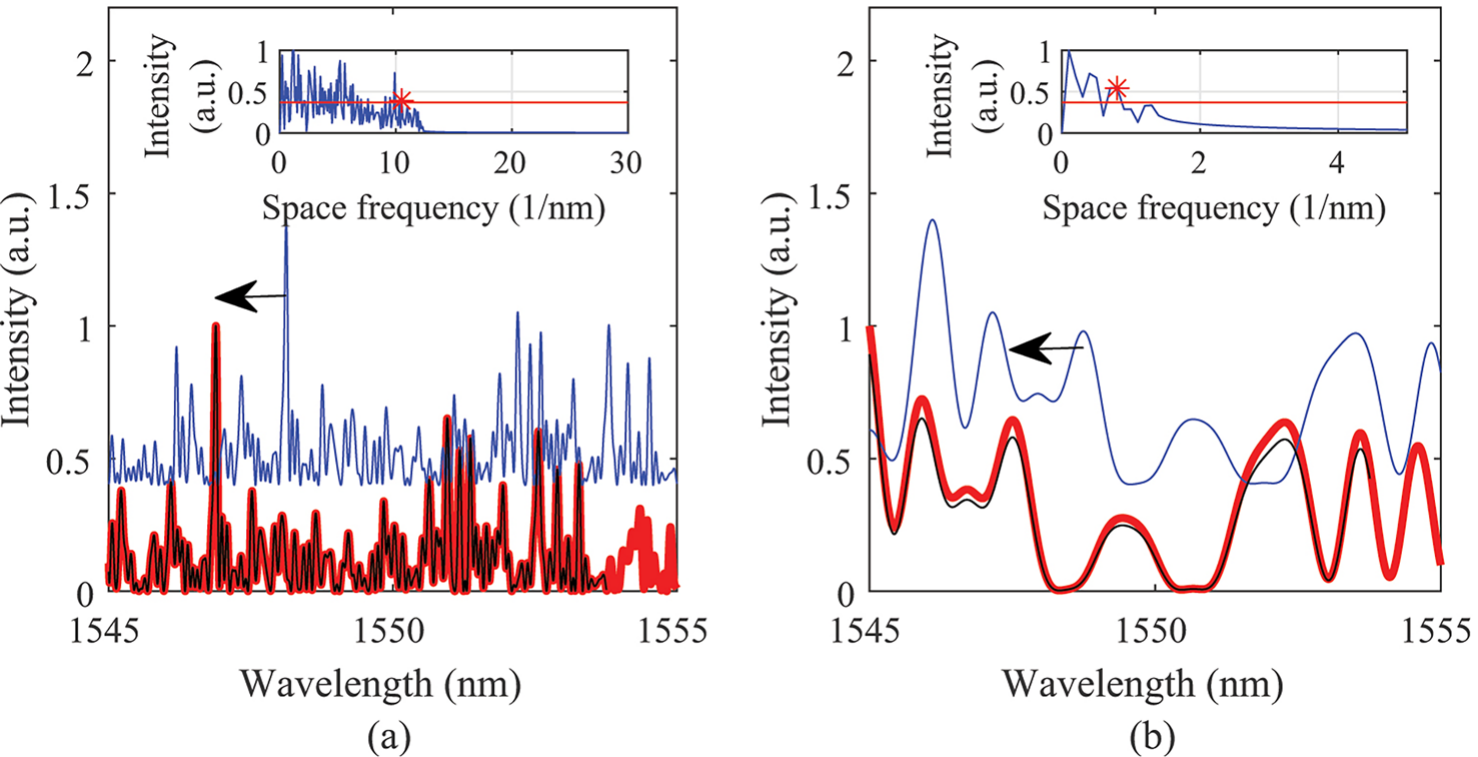
The spectra and the space frequency information under 100 με. (**a**) A case for 200 nm gold NP; (**b**) A case for 400 nm gold NP.

Figure 5 shows two cases of different size gold NPs with different volume ratio in different gauge lengths. Note: the spectra have been normalised by the maximum values. Figure 5a shows the case of 200 nm gold NP with 100 NPs in 1.0690 cm gauge length. In this case, the single scattering length is 41.4476 cm and the concentration of gold NP is $1.3198 \times {10{^8}} \,\hbox {mL}{^{-1}}$. The gauge length is smaller than the single scattering length. Therefore, the major components of the light scattered in the gauge length is single scattered light. Figure 5b shows the case of 400 nm gold NP with 100 NPs in 0.1069 cm gauge length, whose single scattering length is 0.2021 cm and for a concentration of gold NP is $1.3198 \times {10{^9}} \,\hbox {mL}{^{-1}}$. The red lines shown in Fig. 5a,b are the spectra under strain 0. The blue lines are the spectra under 100 με. It can be seen that there is a red shift of the spectra under 100 με. The red shift values are about 124 pm for both cases. To compare the original spectra and the spectra under the spectral shift, the spectra with black lines show the spectra with -124 pm shift. The spectra after the translational movement match the original spectra well in Fig. 5a,b. The shift directions are labelled with black arrows. There are slightly more differences between the spectra of red lines and black lines. One reason for the differences is that the intensity of the backscattered light by the NPs is influenced by the RI change of the optical fibre.

Comparing Fig. 5a,b, it can be seen that the density of the spectra are different. There are more spectral fluctuations within the wavelength range for Fig. 5a than the spectral fluctuation in Fig. 5b. The spectra fluctuation numbers are different for different sizes of NPs, volume ratios and gauge lengths.

In order to evaluate the spectral density quantitatively, the spectra were transferred to the wavenumber domain (space frequency) by Fourier transform and then by setting a threshold to obtain a period number for evaluating the spectral density. The period number was defined as the threshold space frequency multiplied the wavelength range, so it is a dimensionless value to evaluate the spectra fluctuation property.

The embedded graphs in Fig. 5 show the results of the spectra after Fourier transform under strain 0 for evaluating the spectral density. The spectra with averaged intensity value of 0 in the wavelength domain were transferred to the wavenumber domain. The threshold of 1/*e* was set to evaluate the spectra fluctuation property. The red stars are the first points that meet the requirement of being above the threshold from the high space-frequency side. The period number of the spectra in Fig. 5a is larger than the spectra in Fig. 5b which is consistent with the results seen from the wavelength domain directly.

**Figure 6:**
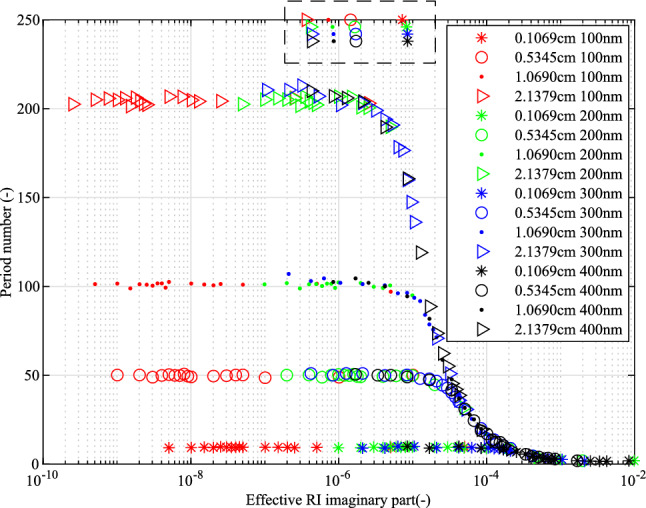
Period number with volume ratio ratios for different sizes of NPs (100 nm in red, 200 nm in green, 300 nm in blue and 400 nm in black) with different gauge lengths (0.1069 cm with star, 0.5345 cm with circle, 1.0690 cm with triangle and 2.1379 cm with point). The patterns in the dash rectangular frame are only used show the maximum values of the imaginary part of effective RI based on the single scattering restriction.

The period numbers may be influenced by the size of the NPs, the volume ratio of the NPs in the optical fibre and the gauge lengths. The influence of the period number for these parameters was analyzed. Figure 6 shows the period numbers for different imaginary parts of the effective RI. The different imaginary parts of the effective RI correspond to different volume ratios of NPs. One reason for choosing imaginary parts of the effective RI is that the imaginary parts of the effective RI not only consist of the volume ratio but also consist of the property of the materials, for example absorbing NP materials and non-absorbing NP materials. Therefore, the imaginary part of the effective RI is a more universal parameter compared with volume ratio. As shown in Fig. 6, the period number increases when the gauge length increases in the lower imaginary part of the effective RI range (less than about ${2 \times {10{^{-6}}}}$). When the imaginary part of the effective RI range is above this range, the peroid number shrinks to low values. This indicates that the gauge length has a high influence on the spectra fluctuation and that the imaginary part of the effective RI adjusts the period number for high doping concentrations. The size of the NPs shows a low influence on the period number for these four different sizes of NPs, which may indicate the the gauge length and the imaginary part of the effective RI are the major factors for period number so as to the characteristic of the fluctuation density of the backscattering spectra. However, the single scattering restriction limits the doping concentration to low levels, see the maximum values of the imaginary part of effective RI shown in a black dash rectangular frame in Fig. 6. Under single scattering restriction, period number shows the independence of the size of the NPs and the period number only influenced by the gauge lengths. Longer gauge lengths show larger period values.

**Figure 7:**
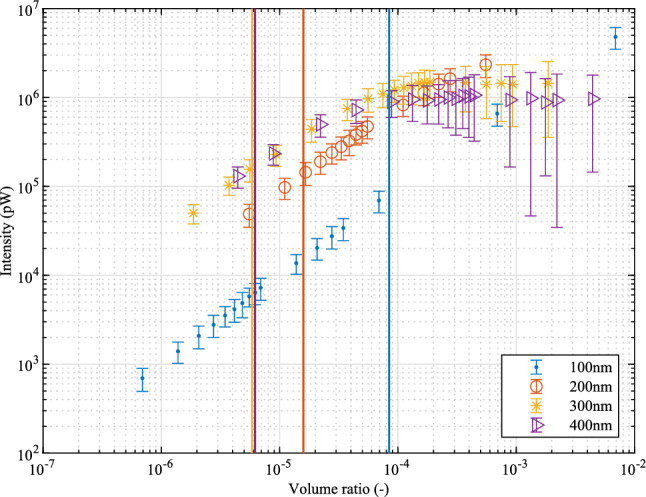
Intensity of backscattered light under different volume ratios of the NPs with 0.1069 cm gauge length. The blue, red, orange and purple lines show the maximum volume ratios in the case of single scattering for 100 nm, 200 nm, 300 nm and 400 nm respectively. (Incident light power ${1} \,\hbox {mW}$).

The intensities of the spectra were normalised in the previous analysis. The intensity of the backscattered light is also a characteristic of the spectra. In order to evaluate the backscattered light intensity, Fig. 7 was plotted for different volume ratios of gold NPs. The reason for choosing volume ratio as the variable is that the results can be compared with the previous results^21^. Because of the random seeding of the NPs in the optical fibre, the intensity of the spectra is not a constant. Therefore, the results are plotted with error bars. The error bar shows the maximum intensities and minimum intensities by 100 times averaging by Monte Carlo seeding in a gauge length of 0.1069 cm optical fibre. The blue line, red line, yellow line and pink line show the maximum volume ratios for single scattering for 100 nm, 200 nm, 300 nm and 400 nm respectively. Although larger size NPs generally have a higher intensity for backscattering, the single scattering requirement restricts the volume ratio. Therefore, it can be seen that 200 nm size gold NPs have similar backscattering intensity compared with larger sizes (300 nm and 400 nm sizes gold NPs) cases, which is consistent with the previous work for optimisation of gold NPs in the optical fibres^21^. It can also be seen that although increasing the volume ratio over the single scattering limitation may increase the backscattered light, the increase is reduced for high volume ratios. This may be caused by the absorbing property of the gold. High concentrations of gold NPs may shorten the effective gauge length and only the scattered light in the front of the gauge length would have an influence on the backscattered light spectra.

**Figure 8:**
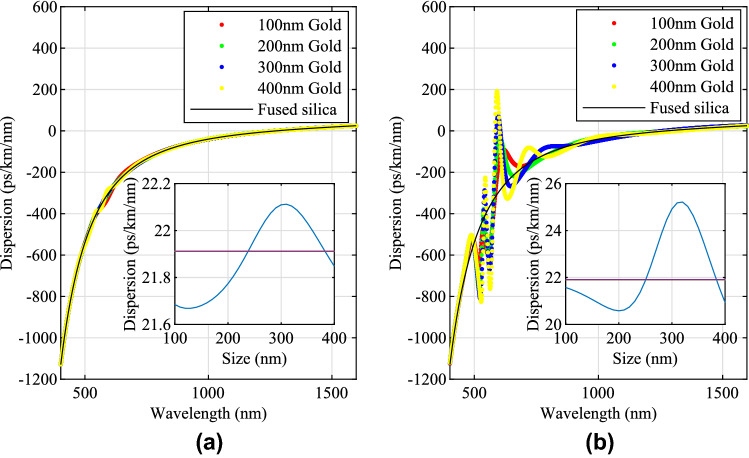
The material dispersion of gold NP-doped optical fibres. (**a**) The cases of material dispersion of 1 cm length gold NP-doped optical fibres with the maximum concentrations for single scattering restriction. The red dots show the case for 100 nm size gold NPs. The green dots show the case for 200 nm size gold NPs. The blue dots show the case for 300 nm size gold NPs. The yellow dots show the case for 400 nm size gold NPs. The black lines show a reference of material dispersion of fused silica. The embedded graph shows the dispersion of 1 cm length gold NP-doped optical fibres with the maximum concentrations for single scattering restriction for different sizes of gold NPs from 100 to 400 nm at 1550 nm incident light in blue lines. The purple lines show the dispersion of fused silica at 1550 nm. (**b**) The cases of material dispersion of 1 cm length gold NP-doped optical fibres with a fixed volume ratio of ${1 \times {10{^{-4}}}}$. The red, green, blue, yellow dots show the case for 100 nm, 200 nm, 300 nm and 400 nm gold respectively. The black lines show the dispersion of fused silica. The embedded graph shows the dispersion of 1 cm length gold NP-doped optical fibres with volume ratio of ${1 \times {10{^{-4}}}}$ for different sizes of gold NPs at 1550 nm incident light in blue lines. The purple lines show the dispersion of fused silica at 1550 nm.

Doping gold NPs into the optical fibre has an influence on the light dispersion in the optical fibre. The RI of fused silica^26^ was used as the material for optical fibre. By doping gold NPs, the dispersion was tuned. The group velocity dispersion ($D_{\lambda }=-\frac{\lambda }{c}\frac{\mathrm {d}^{2} \Re {n_{eff}} }{\mathrm {d} \lambda ^{2}}$) was used to evaluate the material dispersion, where *c* is the velocity of light in a vacuum and $\Re {n_{eff}}$ is the real part of the effective RI of optical fibre. Then, the material dispersion of gold NP-doped optical fibre is shown in Fig. 8.

Figure 8a shows the cases of material dispersion of 1 cm length gold NP-doped optical fibres with the maximum concentrations for single scattering restriction at 1550 nm. The black lines show the case of fused silica (without doping NPs) as a reference. The red, green, blue and yellow dots show the cases for 100 nm, 200 nm, 300 nm and 400 nm gold NPs respectively. It can be seen that dispersion deviations occur between the dispersion curve of fused silica and NP-doped fibres within the spectral range from 400 to 1600 nm in Fig. 8a. An embedded graph shows the cases of the material dispersion with different sizes of gold NPs (from 100 to 400 nm with an interval of 10 nm) at 1550 nm incident light in blue lines. The purple line shows the dispersion of fused silica as a reference. The dispersion of the material was tuned by doping NPs.

Figure 8b shows the cases of material dispersion of 1 cm length gold NP-doped optical fibres with a fixed higher volume ratio (${1 \times {10{^{-4}}}}$). It can be seen that in the visible light range, the dispersion has been tuned dramatically and for some wavelengths the dispersion can be zero. It indicates that zero dispersion may be achieved by doping gold NPs with specific sizes and concentrations for some wavelengths. The embedded graph in Fig. 8b shows the cases of the material dispersion with different sizes of gold NPs at 1550 nm incident light in blue lines. The purple line shows the dispersion of fused silica. It can be seen the shape of the dispersion curve has been tuned intensely compared with the cases in the embedded graph in Fig. 8a. due to the increasing of the concentration of NPs.

## Conclusion

NP doped optical fibres have similar spectral shift behaviour under strain compared with FBGs or distributed strain sensing based on Rayleigh scattering. The characteristics of the spectra (spectra fluctuation and backscattered intensity) were investigated. The imaginary part of the effective RI has a high influence on the period number of the spectra. Gauge length also showed a correlation to the period number. However, the size of the NPs did not show a relationship with the period number from 100 size to 400 nm size gold NPs range. The intensity of the backscattered light is restricted by the single scattering volume ratio. Although increasing the volume ratio further may increase the backscattered light intensity, the absorbing property of the material restricts the tendency of the intensity to increase. These theoretical results may promote the future design in engineering of NP doped fibre sensors.

## Method

**Figure 9:**
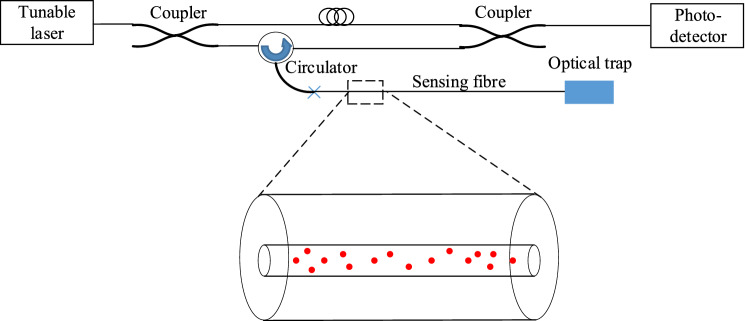
Experimental setup. The dash rectangular zone shows a gauge length which has been enlarged. Red dots indicate randomly positioned gold NPs in the core of the optical fibre. The core of the optical fibre is surrounded by outer layers.

Figure 9 shows a simplified structure of the experimental setup of a typical distributed fibre optic sensing based on optical frequency domain reflectometry. Light emitted by a tunable laser is coupled into a Mach-Zehnder interferometer. One part of the interferometer includes a sensing fibre containing NPs inside of the core of the optical fibre. The backscattered light from the NPs in the sensing fibre will interfere with the light transmitted in the reference arm, which will then be detected by a photodetector. The light backscattered at different positions along the optical fibre will generate a beat signal with the reference signal which can be used for local strain detection with millimetre resolution. By demodulating the time-series signal detected by the photodetector with Fourier transform to obtain spectra information and comparing the spectra obtained before and after strain change, the strain can be obtained. Generally, the optical setup includes an additional interferometer to compensate for the non-linearity of the tunable laser and there is also a polarized beam splitter before the photodetectors to compensate for the polarization changes in the optical fibre. For simplification, Fig. 9 only shows the basic components for the strain detection.

In the simulation procedure, 100 nm, 200 nm, 300 nm and 400 nm size spherical gold NPs were chosen as the typical large size gold NPs used for simulations. The volume ratios chosen were from about $10^{-10}$ to about $10^{-2}$ with different gauge lengths (0.1069 cm, 0.5345 cm, 1.0690 cm, and 2.1379 cm which corresponds to 2000 times, 10,000 times, 20,000 times and 40,000 times $\Lambda$ for a period length of a FBGs at effective RI of 1.45). The simulation procedure can be described as: (1) Choosing a specific size of gold NP which is used for simulation; (2) Choosing a specific gauge length; (3) Seeding an integer number of NPs in the given gauge length. The seeding process is a Monte Carlo seeding and the spatial distribution of the NPs follows a uniform distribution within the gauge length; (4) The corresponding volume ratio of gold NPs to the optical fibre and the concentration of the gold NPs can be calculated. Applying the single scattering assumption in the gauges lengths ($l<1/cC_{ext}$) where *c* is the concentration of the NPs in the core of the fibre and $C_{ext}$ is the extinction cross section for the NP, the maximum volume ratios can be obtained; (5) The backscattered light signal can be obtained in the wavelength domain by calculating the square of Eq. (15). *K* is approximated as 1 in the simulation; (6) By Fourier transform, the signal was transferred to the wavenumber domain to compare the spectral density characteristics with a threshold of 1/*e* of the maximum. The wavenumber at the threshold was recorded as the parameter to characterise the fluctuation of the spectra. The period number can be obtained by the wavelength range multiplying the wavenumber at the threshold.

## Data Availability

The datasets generated and analysed during the current study are available in the 4TU.ResearchData repository, 10.4121/20013470.
